# Life beyond a jar: Effects of tank size and furnishings on the behaviour and welfare of Siamese fighting fish (*Betta splendens*)

**DOI:** 10.1017/awf.2024.67

**Published:** 2024-12-23

**Authors:** Naomi Clark-Shen, Juliette Tariel-Adam, Anya Gajanur, Culum Brown

**Affiliations:** 1 Independent researcher, Singapore; 2 Macquarie University, School of Natural Sciences, Australia; 3 Virginia Institute of Marine Science, William & Mary, USA

**Keywords:** animal welfare, aquarium, *Betta splendens*, ornamental fish, pet, sentience

## Abstract

Globally, Siamese fighting fish (*Betta splendens*) continue to be sold and kept in small, barren jars or tanks, with little concern for their welfare. This study aimed to examine the impact of housing size and furnishings (i.e. live plants, refuges) on the behaviour of Siamese fighting fish, to understand optimal tank conditions. Thirteen male Siamese fighting fish were rotated between five different housing conditions: ‘jar’ (1.5 L); ‘small’ (3.3 L); ‘medium’ (5.6 L); ‘large’ (19.3 L); and ‘large-barren’ (19.3 L). All tanks had gravel and furnishings, except the large-barren tank which was devoid of these. Overall, tank size influenced behaviour. Fish were significantly more active and spent significantly less time resting and performing ‘abnormal’ behaviours (hovering and stereotypic swimming), in the large tank compared to the smaller tanks. Tank furnishings also influenced behaviour. Fishes in the large-barren tank performed more ‘abnormal’ behaviours (hovering, stereotypic swimming, interaction with the walls), compared to the large tank which had furnishings. These results suggest that the small, barren jars and tanks that Siamese fighting fish are often housed in are detrimental to their welfare, and larger, furnished tanks are more optimal. Behavioural variations were observed between the fish in this study, highlighting individual fish personality. We recommend a minimum tank size of 5.6 L for the display and sale of Siamese fighting fish, and tanks larger than this for keeping Siamese fighting fish at home. All tanks should contain gravel, live plants and refuges.

## Introduction

Fishes are rarely afforded the same level of compassion, welfare and legislation as other vertebrates (Brown 2015). However, there is growing recognition that fishes possess impressive memory and cognitive abilities (White & Brown 2014; Brown 2015), social intelligence (Oliveira *et al.* 1998; Bshary 2011), individual personalities (Byrnes & Brown 2016), and the capacity to feel pain (Sneddon 2003; Reilly *et al.* 2008). With increasing recognition of their sentience (Brown 2015), concerns for their welfare are growing (Berlinghieri *et al.* 2021), as is the need to improve their treatment across industries – from capture fisheries (Veldhuizen *et al.* 2018), to aquaculture (Kleiber *et al.* 2023), in laboratories (Smith 2014), and as pets (Sermwatanakul 2019).

Fishes are the most numerous pet in the world (Brown 2019), with an estimated value of up to US$20 billion, and over 6,000 species traded internationally (Moorhead & Zeng 2010; Novak *et al.* 2020). The Siamese fighting fish (*Betta splendens*) is one of the most popular pet fishes. Originating from rivers in Southeast Asia, they have been bred for exquisite colours and elongated fins (Monvises *et al.* 2009). Thailand is one of the biggest suppliers of Siamese fighting fish; in 2018, there were over 1,000 Siamese fighting fish farms in Thailand with exports numbering over 20 million individual fish (Sermwatanakul 2019). The USA, China, Singapore, France and Iran are top importers (Sermwatanakul 2019). Siamese fighting fish are typically sold and then kept in jars or small ‘betta vases’, often with no tank accessories, both of which are acknowledged as potential welfare concerns (Sermwatanakul 2019). However, recommendations for appropriate tank size are vague. For example, one Siamese fighting fish handbook states that “*a large aquarium is preferred over a small one*” (Goldstein 2004), while another source states “a minimum of four litres”. These suggested conditions arise out of concerns over water quality, not behavioural indicators of poor welfare (Pleeging & Moons 2017). Singapore’s ‘Pet Shop Licence Conditions (5) Display and Sale of Fancy Fish’ Act states “*Fishes must be kept in tanks of adequate size*” (Animal & Veterinary Services 2023) but does not state what this is and still allows Siamese fighting fish to be sold in small jars. Although one study has shown that Siamese fighting fish swim more in larger tanks compared to smaller ones (Oldfield & Murphy 2024), the general lack of research and attention in this area has made is difficult to change consumer behaviour and policy to improve welfare.

Behaviour is recognised as a key indicator of an animal’s emotional state in captivity (Dawkins 2003). For example, abnormal repetitive behaviours, such as stereotypic pacing, are defined as functionless and may represent an inability to cope with the captive environment, while increased behavioural diversity is a potentially positive indicator of welfare (Miller *et al.* 2020). Studies on captive animals reveal that in general, more naturalistic living conditions promote the freedom to express natural behaviours, which can contribute to greater welfare (Kleiman *et al.* 2010; Alligood *et al.* 2015). Compared to terrestrial animals in zoos, there are fewer studies examining the welfare of fishes in captivity, however those that have been conducted reveal parallels (Toni *et al.* 2018; Barreto *et al.* 2020). A study on black rockfish (*Sebastes melanops*), for example, found that an absence of plant and structural enrichment significantly increased stress, as determined through higher cortisol levels (Zhang *et al.* 2020). In a study on sharks, rays and teleost fishes in an aquarium, enhanced environmental complexity and reduced visitor exposure resulted in increased natural behaviours and decreased abnormal repetitive behaviours (Lawrence *et al.* 2021). Similarly, small tanks triggered stereotypy and reduced boldness in zebrafish (*Danio rerio*) (Abudusaimaiti *et al.* 2020). Other studies have found that big enclosures make fish swim faster (Tang & Boisclair 1993), grow better and result in higher survival rates (Jha *et al.* 2006). Collectively these studies suggest that improved housing conditions have positive outcomes on behaviour and welfare. However, different species of fish have unique behavioural repertoires, so species-specific assessments to determine optimal housing are preferential (Smith 2023).

This study aimed to examine the impact of housing size and furnishings (e.g. plants, refuges) on the behaviour of Siamese fighting fish. A greater understanding of Siamese fighting fish behaviour in relation to tank size and enrichment (i.e. furnishings) can help to guide stakeholders (e.g. sellers, buyers, policy-makers) toward improved welfare for these animals. Specifically, the outcomes will guide recommended minimum tank size to promote better welfare for Siamese fighting fish. The study was conducted in Singapore, which has been the number one exporter of ornamental fishes since the 1980s, and where fish-keeping is a popular local hobby (Hua Yue 2019), with Siamese fighting fish sold in numerous pet shops and fish shops. Thus, while Singapore is a suitable country to conduct this research and drive change, the impacts of this research are global.

## Materials and methods

### Ethical considerations

As this study was not conducted within a university, formal animal ethics approval was not attained, however, we followed the recommendations as outlined by the Association for the Study of Animal Behaviour (ASAB Ethical Committee/ABS Animal Care Committee 2023) and an expert in the field of fish welfare (CB) was consulted throughout. All the tanks used in this study were larger than the small cups/jars in which the fish were sold, thus affording them more space than they had previously been accustomed to. Additionally, a fish shop in Singapore which is known for promoting high welfare for fish-keeping was consulted to ascertain how to monitor water quality (through test kits) and reduce stress (i.e. through use of ‘black water’ and ‘smoothing fluids’). When the study was completed, three fish were adopted by NCS and kept in various tanks > 5.6 L in size and with plants, gravel and hideouts. All the remaining fish (n = 10), were adopted out to friends, or friends-of-friends, with strict conditions regarding tank size and furnishing (> 5.6 L, larger than the ‘medium’ tank used in this experiment, and with plants and gravel). All adopters were asked to send photographs of their tank set-up, and photographs/videos and updates following adoption, to ensure the fish were kept in the tanks that were promised and were doing well. NCS maintained contact with all adopters to provide continuous advice on fish-keeping.

Fifteen male Siamese fighting fish (labelled as ‘Half-moon bettas’) were purchased from an ornamental fish farm in Singapore in December 2021 (seven fish) and January 2022 (eight fish). At the ornamental fish farm from which they were bought, they were displayed, and sold in, barren circular jars (8.5 cm × 11.6 cm; height × width; approximately 900 ml water volume). Following purchase, they were randomly assigned to an experimental housing tank (Table 1), to begin the experimental trial.

**Table 1.** Siamese fighting fish (*Betta splendens*) were housed individually and rotated between five tank treatments during this study. The tank sizes, furnishings and the duration that they were kept in each treatment during the study is presented

Tank	Size (length × width × height) (cm)	Volume of water (L)	Furnishing	Duration in tank (days)
Jar	10 × 10 × 15	1.5	Pebbles and three small surface plants	7
Small	15 × 15 × 15	3.3	Pebbles and one medium plant	7
Medium	22 × 15 × 17	5.6	Pebbles and one medium plant	7
Large	35 × 23 × 24	19.3	Pebbles, one large plant, and one hideout (barrel)	7
Large - barren	35 × 23 × 24	19.3	None	3

### Experimental tank set-up

**Figure 1.**
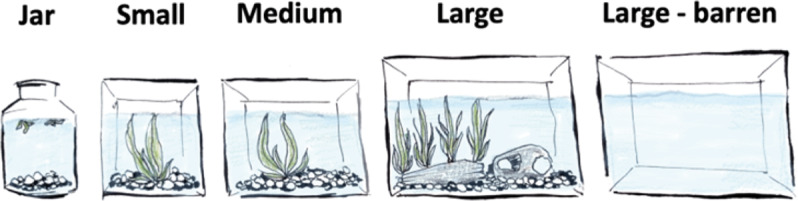
Siamese fighting fish (*Betta splendens*) were housed individually in and rotated between jar (1.5 L; three small surface plants and pebbles), small (3.3 L; one medium plant and pebbles), medium (5.6 L; one medium plant and pebbles), large (19.3 L; one large plant, pebbles and one barrel for refuge), and large-barren (19.3 L; no plants or furnishings).

Five tanks of varying sizes and furnishings were used: ‘jar’; ‘small’; ‘medium’; ‘large’; and ‘large-barren’ (Table 1, Figure 1). To prevent fish getting distracted by human activity, a white sheet was placed about 1 m in front of the tanks and held in place throughout the experiment. Small square flaps were cut in the sheet to allow a camera (iPhone) to record the tanks on ‘recording’ days. Outside of recording days, the square flaps were pinned back in place to ensure there were no gaps in the sheet. Fish only experienced interaction with people during feeding and cleaning (see *Tank maintenance*). All tanks were covered (using grey industrial tape) at both ends to prevent fish in adjacent tanks from seeing each other. Of the 15 fish, seven had a filter in their tank during the small, medium and large trials, while eight did not have a filter during any of the tank trials. The filter was not used for all trials since typically Siamese fighting fish are not housed with filters.

### Rotation of fish

Fish stayed in each of the four tanks that had furnishings (jar, small, medium, large; Table 1, Figure 1) for seven days to allow fish to acclimate, with filming taking place on the seventh day. Fish were then rotated among these four tanks until they had experienced all of them. Rotation order between tanks was generally as follows: those in jar and medium were swapped, and those in large and small were swapped. Fish were then rotated to the other tanks they had not experienced and swapped in the same manner. This order of rotation was chosen to minimise a drastic downgrade in condition (e.g. from large to jar) which may have induced heightened stress. Once fish had spent seven days in each of these four tanks, they were rotated into the barren tank (large-barren), but to reduce potential risk of stress or mortality from the sudden change in tank condition (i.e. from having furnishings to having none, which can also affect water quality), fish only stayed in this tank for three days, with filming on the third day. When fish were rotated into each of the five tanks (jar, small, medium, large, large-barren) they were observed for 5 min and if any fish reacted in a way that indicated an inability to cope (e.g. they were suffering; referring to unpleasant mental states such as fear or pain; Brown 2015) they were given an ‘exemption’ from that particular tank (e.g. a total skip of that tank) and placed in a ‘holding’ tank (which fell between the sizes of small and medium, and contained pebbles and a plant) for seven days until they could be rotated back into the trial. Table 2 outlines the order in which each fish experienced each tank.

**Table 2.** The order in which the Siamese fighting fish (*Betta splendens*; n = 15) were rotated between five tank treatments during this study (Jar, Small, Medium, Large, and Large-Barren). 1 = first, 2 = second, 3 = third, 4 = fourth, 5 = fifth. ‘EX’ is used where fish were given an exemption from a tank due to exhibiting what was perceived to be a potential inability to cope, ‘(F)’ indicates if a filter was present in the tank, and ‘(D)’ is used if and when a fish passed away

Fish	Jar	Small	Medium	Large	Large-barren	Observations
Kraken	1	3	2 (F)	4 (F)	5 (F)	
Pegasus	1	3	2	4	5	
Makara	1	3 (D)	2			Water quality test revealed no issues. He was in general lethargic from the start.
Orang Pendek	2	4	1 (F)	3 (F)	5 (F)	
Phoenix	2	4	1 (F)	3 (F)	5 (F)	
Wizard	2	1	3 (F)	4 (F)	5 (F)	
Merlion	2	4	1	3	EX	Extremely skittish, appeared panicked in the barren tank and exhibited fear (e.g. fast swimming to hide behind objects) in previous tanks during feeding.
Kinara	2	4	1	3	5	
Elf	3	2	4	1	5	
Naga	3	2	4	1	5	
Fairy	4	1	3 (F)	2 (F)	5 (F)	
Ghost	4	1	3 (F)	2 (F)	5 (F)	
Goblin	4	1	3	2	5	
Dragon	EX	2	3 (F)	1 (F)	5 (F)	Exhibited behaviours indicating poor welfare (laying on his back, struggling to swim to the surface for air). He was the largest fish in the sample.
Hercules		1		2 (D)		Water quality test revealed no issues. He exhibited symptoms of fin rot from the point of purchase.

### Recording behaviours

On the day of recording (day seven in jar, small, medium, large, and day three in large-barren), fish were recorded for a 10-min period between 0700–0730h (dependent on when the sun had risen and there was sufficient daylight), and again at 1000, 1400 and 1800h, resulting in a total of 40-min footage per fish per tank. These recordings were then subsequently reviewed, and ‘continuous’ behavioural observations were recorded (Table 3).

**Table 3.** Behavioural categories recorded for Siamese fighting fish (*Betta splendens*; n = 15) rotated between five tank treatments. The behavioural categories chosen were based on observed behaviours shown during the recordings themself. The total duration of each behaviour was recorded in seconds

Behaviour	Description
Resting	Motionless (fins not moving). Body in contact with a surface (ground, object, plant, side of tank, water surface).
Hovering	‘Hanging’ motionless in the water column, body not in contact with any surface. Pectoral fins typically moving.
Sinking or floating	Not actively swimming or moving fins, but fish sunk downward or floated upward.
Swimming	Irregular, non-stereotypic, varied routes and speeds.
Stereotypical swimming	Repetitive movements with no clear objective or outcome - e.g. pacing back and forth, circling. Considered once a movement has been performed 3 times in a row. Pacing is ‘broken’ if fish deviates significantly off course.
Interaction with the walls	Fish makes contact with a surface while moving, either the ground or the side walls. No time limit (e.g. doesn’t have to occur for 3 seconds to count). Does not include resting even if fish resting against a surface.
Out of view	Fish behaviour cannot be observed.
Nest building	Fish creates bubbles on the water surface and either hovers underneath or continuously puts mouth to water surface to build a nest.
Foraging	Fish appears to be searching for, then ‘nipping’ at small food items on the floor or on/under items.
Unsure	Behaviour cannot be confidently assigned into one of the above categories.

### Tank maintenance

Fish were fed twice per day, at 0800 and 1820h. Larger fish were given six pellets per feeding and smaller fish were given four pellets per feeding. The jar, small, medium, and large tanks underwent a full water change on the seventh day when fish were rotated, but pebbles were not rinsed through as the build-up of bacteria in these pebbles can help maintain water stability. The large-barren tanks were given a full water change on the third day, when fish were rotated. All tank water was replaced with water that had been pre-mixed in a bucket with ‘black water’, which acts as an anti-chlorine, and ‘water smoother’, which softens water (9.56 ml black water and 86 ml of smoothing fluid pre-mixed per 43-L bucket). Fish were rotated into their newly cleaned tanks via a small scoop that contained water (< 1 L of water) from their previous tank. No artificial lights were used, and at night the room lights were switched off, allowing for near-total darkness. A water-quality monitoring kit (Pro JBL Aquatest Easy 7 in 1, JBL, Neuhofen, Germany) was used in each tank, on the day before recording, to test for ammonia, nitrate, nitrogen dioxide, GH (hardiness), alkalinity, pH, carbon dioxide and chlorine gas, all of which can affect fish behaviour and survival.

### Data analysis

While 15 fish started the trial only 13 completed it (see Table 2) and statistical analyses were only performed on data pertaining to these 13 fish. Statistical analyses were carried out to test the effect of tank on fish behaviour, while controlling for fish identity, tank order (Table 2), time of the day and presence of filter. Times where fish were ‘out-of-view’ or if the type of behaviour could not be determined (‘unsure’), were not included in the analysis (Table 3). The times spent ‘resting’, ‘hovering’, ‘sinking or floating’, ‘swimming’, ‘stereotypical swimming’, ‘interaction with the walls’, ‘nest building’ and ‘foraging’ during a trial were first analysed with a Principal Component Analysis (PCA). The PCA was performed: (1) to reduce the number of behaviours to analyse; (2) to look at the correlation between behaviours; and (3) to assess which behaviour(s) were the most important to explain the behavioural variability between trials. The PCA did not help to reduce the number of behaviours to analyse. Only the first four components were kept as they had an eigenvalue > 1 and explained 70% of the total variance. However, the fourth component was not readily associated with the initial behaviours and therefore cannot be biologically interpreted (Figure 3). After excluding the fourth component, the first three components collectively explained only 57% of the variance. We thus decided to perform individual linear models on each behaviour rather than on these first four components. ‘Sinking or floating’ did not not undergo further analysis since it was explaining less than 2% of the total behavioural variance.

**Figure 2.**
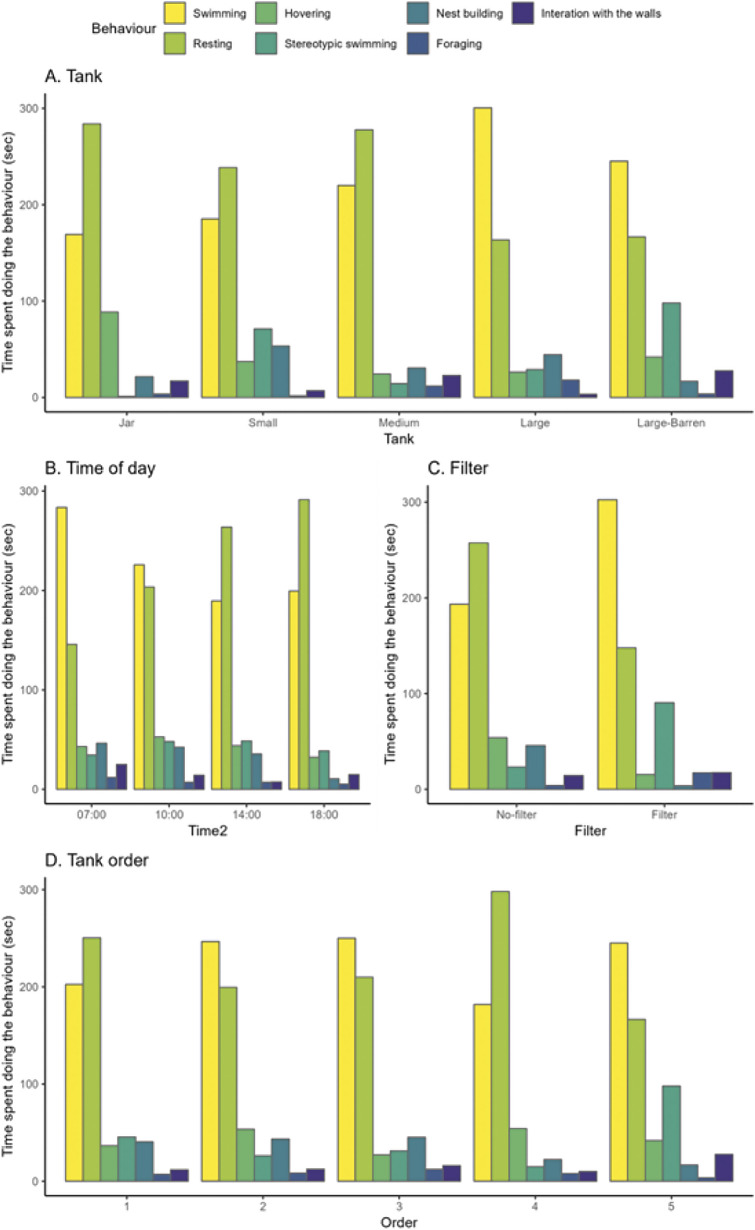
Behaviours observed (A) in each of the five tank treatments (jar, small, medium, large, and large-barren), (B) across different times, (C) with and without a filter in the tank and (D) in the order in which fish were rotated into the tank. Each bar represents an average time spent displaying behaviours by the Siamese fighting fish (*Betta splendens*; n = 13) which completed the rotation between the five tank trials.

**Figure 3.**
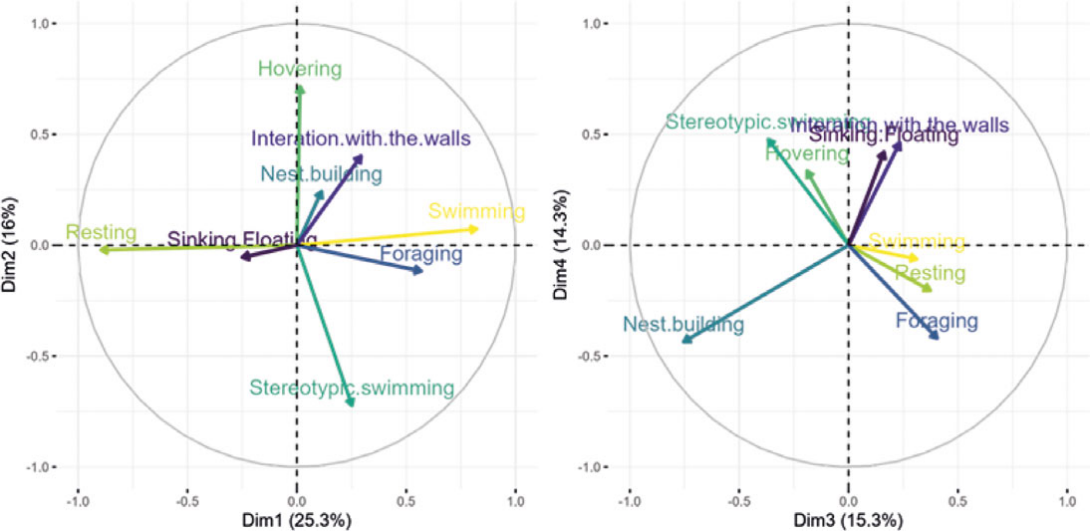
Results of Principle Component Analysis for the times that the Siamese fighting fish (*Betta splendens*; n = 13) spent performing each behaviour across the five tank treatments (jar, small, medium, large, and large-barren).

The time spent swimming and the time spent resting were analysed separately with a linear mixed model (LMM) including tank (factorial variable with five levels: jar, small, medium, large, large-barren), time of the day (factorial variable with 4 levels: 0700, 1000, 1400, 1800h), filter (factorial variable with 2 levels: presence of filter, absence of filter) and tank order (factorial variable with five levels: 1, 2, 3, 4, 5) as fixed effects and fish identity (factorial variable with 13 levels: Dragon, Elf, Fairy, Ghost, Goblin, Kinara, Kraken, Merlion, Naga, Orange Pendek, Pegasus, Phoenix, Wizard) as a random effect. Tank order was a categorical rather than a continuous variable as the relationship between time spent swimming/resting and tank order was not linear. The models were rank deficient for tank and tank order: The level ‘5’ of tank order was confounded with the level large-barren of tank as large-barren was the fifth tank in which fish went into for all fish (Table 2). This means that we could not statistically disentangle the effect of ‘barren’ from the effect of the fifth tank order in the analysis.

The times spent hovering, stereotypically swimming, interacting with the surface, nest building or foraging did not follow a normal distribution as there were too many zeros (i.e. trials where the fish did not perform this behaviour at all). These behaviours were thus analysed as occurrences/binary variables: (1) if a fish performed the behaviour during the trial, (0) if not. The occurrences of hovering/stereotypically swimming/interacting with the surface/nest building/foraging during a trial were separately analysed with a generalised linear mixed model (GLMM) with tank, time of the day, filter and tank order as fixed effects and fish identity as random effect. As there were still a lot of trials during which fish hovered (204 out of 252 trials), an LMM on the time spent hovering was conducted in addition to the GLMM on the occurrence of hovering. The LMM included only the trials during which the fish hovered. It had tank, time of the day and filter as fixed effects and fish identity as random effect. The model was fitted with weighted least squares using the package nlme to account for the residual variance increasing with the fitted hovering time values. For this reason, it was not possible to estimate the variance explained by the fish random effect or the repeatability. In addition, the nlme package does not allow for rank deficiency so we did not include tank order in the hovering LMM. Included or not, the tank order did not change the significance of the other fixed effects in the previous LMMs and GLMMs.

Additional analysis was performed to test whether fish had a preference for resting on furnishings (plants, hideout barrel or filter) over the floor or at the water surface. The resting place was scored whenever a fish was resting and categorised as resting on the floor, water surface, plants, in or on hideout barrel and filter. The analysis was restricted to the small, medium and large tanks because they were the only tanks with furnishing inside. Plants, filter and hideout barrel were concatenated into one category ‘furnishings’ to have the same three categories for all tanks (i.e. floor, furnishings and water surface) as the hideout barrel was specific to the large tank and filter was specific to the tanks with filter inside. The analysis included 152 trials during which the fish rested at least 1 s. A multinomial regression model was employed to analyse the proportions of resting time in the three places. This model is the extension of the binomial logit model to more than two categories. The response variables were the number of seconds resting on the floor, the number of seconds resting on furnishings and the number of seconds resting on the water surface during the trial. Due to the specifics of the multinomial regression model, tank, order, time, and filter were combined into a single random effect termed ‘experimental setting’. This approach was chosen to avoid testing the difference in resting time between resting places across all levels of tank, order, time, and filter, which was not the primary biological question. The other random effects included were fish and trial (factorial variable with 152 levels corresponding to the trial ID) to account for the non-independence of seconds belonging to the same trial.

Type II F-tests with Kenward and Roger’s method were used to test statistical significance of fixed effects in the LMMs. Likelihood ratio tests (χ^2^) were used to test statistical significance of the fixed effects in the GLMMs and the random effect in both LMMs and GLMMs. For all models, we visually checked normality of residuals and their homoscedasticity across factor levels. We did not remove any outliers. *Post hoc* pair-wise contrasts between estimated means of Tank were performed to test statistically significant differences between tanks using the Holm *P*-value correction for multiple tests. To account for rank deficiency, estimated means of jar, small, medium and large were averaged over the levels of tank order 1–4 while the estimated means of barren were calculated on level 5 of tank order. Confidence intervals of contrasts provided in the *Results* are 95% CI. R version 4.3.1 was used in addition to the following R packages: tidyverse for data manipulation; ggplot2, ggpubr, ggrepel, ggforce, RColorBrewer, virdis for drawing plots; FactoMineR for the PCA; lme4, nlme, lmerTest, emmeans, glmmTMB, fitdistrplus, DHARMa, rptR, performance for the linear models’ estimation and diagnosis.

## Results

### Influence of tank, time and filter on behaviour

Swimming occurred more in the large tank; resting occurred more in the jar and medium tanks; interaction with the walls occurred more in the jar, medium, and barren tanks; stereotypic swimming occurred more in the small and barren tanks; hovering occurred more in the jar and barren tank (Figure 2A, Table 4). Fish were more active in the morning, with swimming occurring more in the morning and resting occurring more in the afternoon (Figure 2B, Table 4). Hovering and nest building occurred more without a filter in the tank, while swimming occurred more with a filter in the tank (Figure 2C, Table 4). The order in which individuals were rotated between tanks (Table 2) had a significant effect for swimming and resting, with fish swimming more, and resting less, during the second and third tank they were rotated into, after accounting for tank size difference (Table 4).

**Table 4.** Results of the linear and generalised mixed models on different behaviours across the five tanks (Jar, Small, Medium, Large, and Large-Barren), time (0700, 1000, 1400 and 1800h), and filter (presence and absence) for the Siamese fighting fish (*Betta splendens*; n = 13) used in this study

ANOVA					
	Fixed effects	F-value	NumDf	DenDf	*P*-value
Swimming	Tank	9.59	4	233.88	<0.001*
	Time	19.61	3	228.04	<0.001*
	Filter	12.52	1	152.69	<0.001*
	Tank order	3.96	3	230.47	<0.001*
	Random effects	Variance	Repeatability	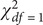	*P*-value
Resting	Fish	1590	0.2 [0.06, 0.37]	33.6	<0.001*
	Residual	5731			
	Fixed effects	F-value	NumDf	DenDf	*P*-value
	Tank	9.91	4	232.15	<0.001*
	Time	21.31	3	228.01	<0.001*
	Filter	0.00	1	224.03	0.99
	Tank order	5.85	3	229.40	<0.001*
	Random effects	Variance	Repeatability	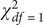	*P*-value
	Fish	8519	0.4 [0.17, 0.59]	77	<0.001*
	Residual	12448			
Hovering	Fixed effects	F-value	NumDf	DenDf	*P*-value
	Tank	18.75	4	183	<0.001*
	Time	3.67	3	183	0.01
	Filter	5.29	1	183	0.02

### Principle Component Analysis (PCA) results

The first principal component (25% of variance) was driven by resting, swimming, and foraging: if fish spent time swimming and foraging, there was little resting. The second component (16% of variance) was driven by hovering, stereotypic swimming, and interaction with walls: if fish spent a lot of time hovering and interacting with walls, there was little stereotypic swimming. The third component (15% of variance) was driven by nest building, followed by a slight influence of foraging, resting and stereotypic swimming: if fish spent time foraging and resting in a trial, they were doing little nest building and stereotypic swimming. However, these last correlations may be driven by trials in the jar as fish rarely engaged in nest building or stereotypic swimming in this tank.

### Commonly observed behaviours

**Figure 4.**
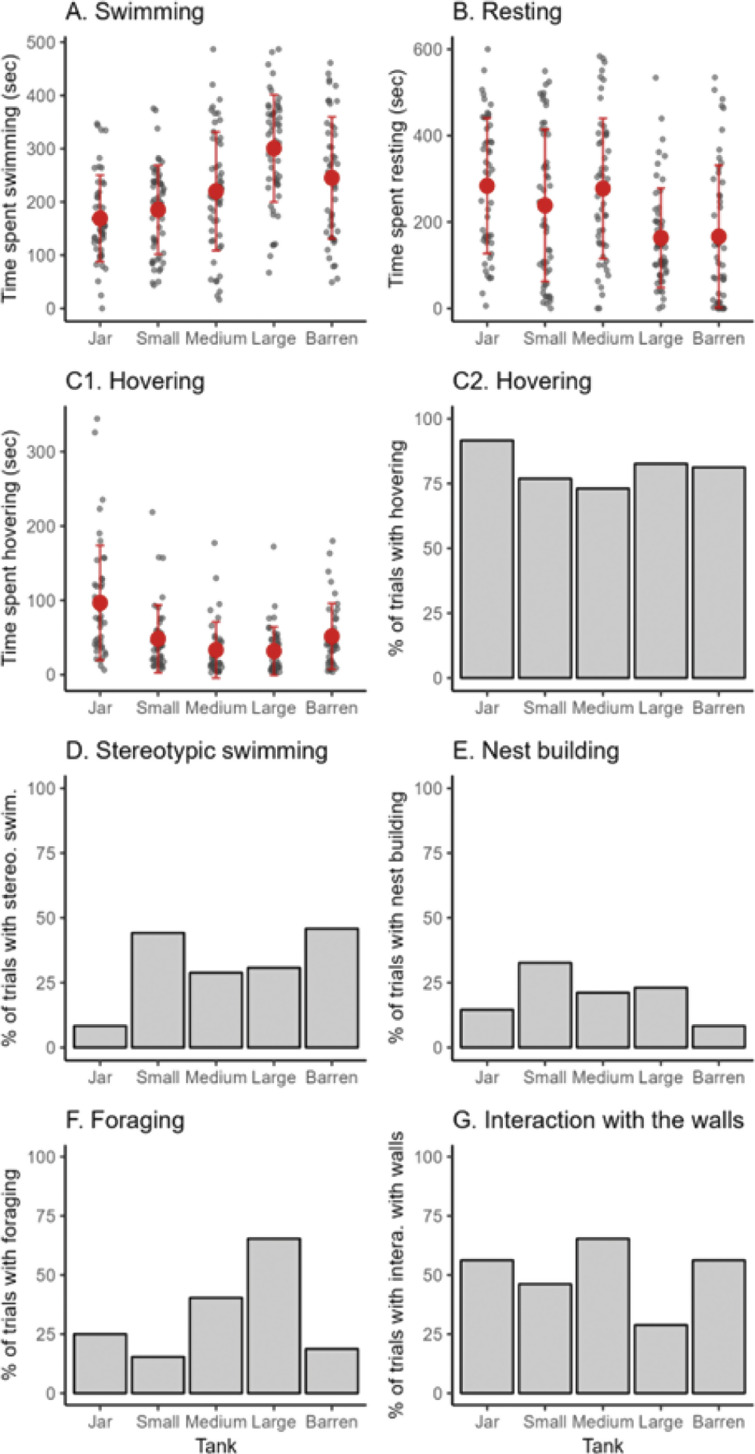
Siamese fighting fish (*Betta splendens*; n = 13) engaging in (A) swimming, (B) resting, (C1, C2) hovering, (D) stereotypic swimming, (E) nest building, (F) foraging and (G) interacting with the surface, across the five tank treatments (jar, small, medium, large, and large-barren). In the graphs, barren refers to large-barren. Each black dot represents the time spent swimming in each trial by each of the Siamese fighting fish. The large red dots represent the average time spent swimming by tank. The scale of the y-axis of each graph differs.

Resting and swimming were the most common behaviours recorded across all treatments. The amount of swimming varied significantly across tank size (Table 4). Individuals in the large tank swam significantly more than in the jar, small, medium, and barren tank (see Table S1; Supplementary material). On average, over each 600-s trial, individuals in the large tank swam 92 s [CI: 46, 138] more than in the jar; 76 s [CI: 30, 121] more than in the small tank; 75 s [CI: 35, 115] more than in the medium tank; and 53 s [CI: 23, 84] more than in the barren tank (Figure 4A).

Congruent with the results for swimming, individuals rested less in the large tank compared to in the jar, small and medium tank (Table 4), but this was only significant between the large tank and the jar and medium tank (Figure 4, Table S1; Supplementary material). Individuals in the large tank rested, on average, 109 s [CI: 40, 178] less than individuals in the jar (over a 600-s trial), and 104 s [CI: 45, 163] less than the medium tank individuals (Figure 4B).

### Less commonly observed behaviours

There was no statistically significant effect of tank on the probability that a fish would hover, however, after excluding the 48 trials where fish did not hover, there was a statistically significant effect of tank on the time spent hovering: fish in the jar hovered 53 s [CI: 20, 85] more than in the small tank; 53 s [CI: 20, 86] more than in the medium tank; and 61 s [CI: 28, 93] more than in the large tank. Individuals in the barren tank hovered significantly more than in the large tank (22 s more [CI: 12, 35]) (Table 4). A remarkably consistent pattern was that for all fish (except Goblin), the time spent hovering reduced between the jar and small tank (Figure 7C).

Stereotypic swimming occurred less in the jar compared to in the small, medium and large tank, but this was only significant between the jar and small tank (Figure 4D, Table 4, Table S1; Supplementary material). Individuals in the small tank, on average, were 33 times [CI: 5, 333] more likely to perform stereotypic swimming than individuals in the jar. Individuals in the barren tanks were 3.4 times [CI: 2, 3] more likely to perform stereotypic swimming than individuals in the large tank. For nesting, there were no statistically significant differences between tanks (Table 4). Looking at the trend, it seemed that nest building was highest in the small tank, and lowest in the jar and barren tanks (Figure 4E).

Foraging occurred significantly more in the large tank compared to in the jar, small, and medium tanks (Figure 4F, Table 4; Table S1; Supplementary material). Individuals in the large tank, on average, were 11 times [CI: 2, 67] more likely to forage than in the jar; 33 times [CI: 4, 200] more than in the small tank; four times [CI: 1, 16] more than in the medium tank; and 15 times [CI: 5, 47] more than in the barren tank.

Interaction with the walls occurred significantly more in the jar and medium tank compared to the large tank (Figure 4G, Table 4, Table S1; Supplementary material). Individuals in the jar, on average, were 4 times [CI: 1, 19.7] more likely to interact with the walls than in the large tank; individuals in the medium tank, on average, were 6 times [CI: 2, 22] more likely to interact with the walls than in the large tank; and individuals in the barren tank, on average, were 3 times [CI: 1, 9] more likely than in the large tank.

### Resting place

**Figure 5.**
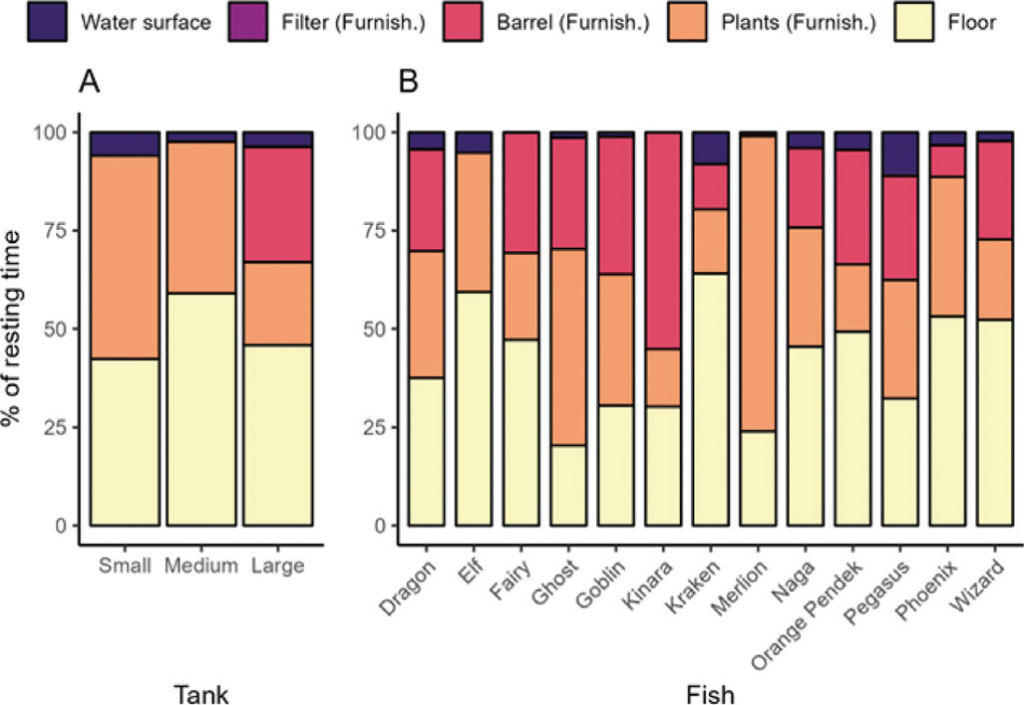
Percentage of total resting time per trial that the Siamese fighting fish (*Betta splendens*; n = 13) spent resting in different places in tanks that had furnishings, by (a) tank size (including small, medium and large tanks only, as these were the only ones with furnishings), and (b) individual fish. The small and medium tanks had plants, while the large tank had plants and a barrel hideout.

When looking at the main three tanks (small, medium, and large) with furnishings (plants, hideout barrel, filter), fish spent more time resting on the floor or on furnishings than on the water surface (Figure 5, Table S2; Supplementary material) but there was no significant difference between floor and furnishings. On average, fish spent 49% of their resting time on the floor, 47% on furnishings and 4% at the water surface. Out of 152 trials during which fish rested, they rested on the floor in 135 trials, on furnishings in 130 trials, and on the water surface in 36 trials. More precisely, in the large tank, they rested 46% on the floor, 29% in or on the hideout barrel, 21% on plants and 4% at the water surface (Figure 5). Fish avoided resting on the filter: fish rested on the filter in only one of the 45 trials where there was a filter inside the aquarium.

### Individual differences

**Figure 6.**
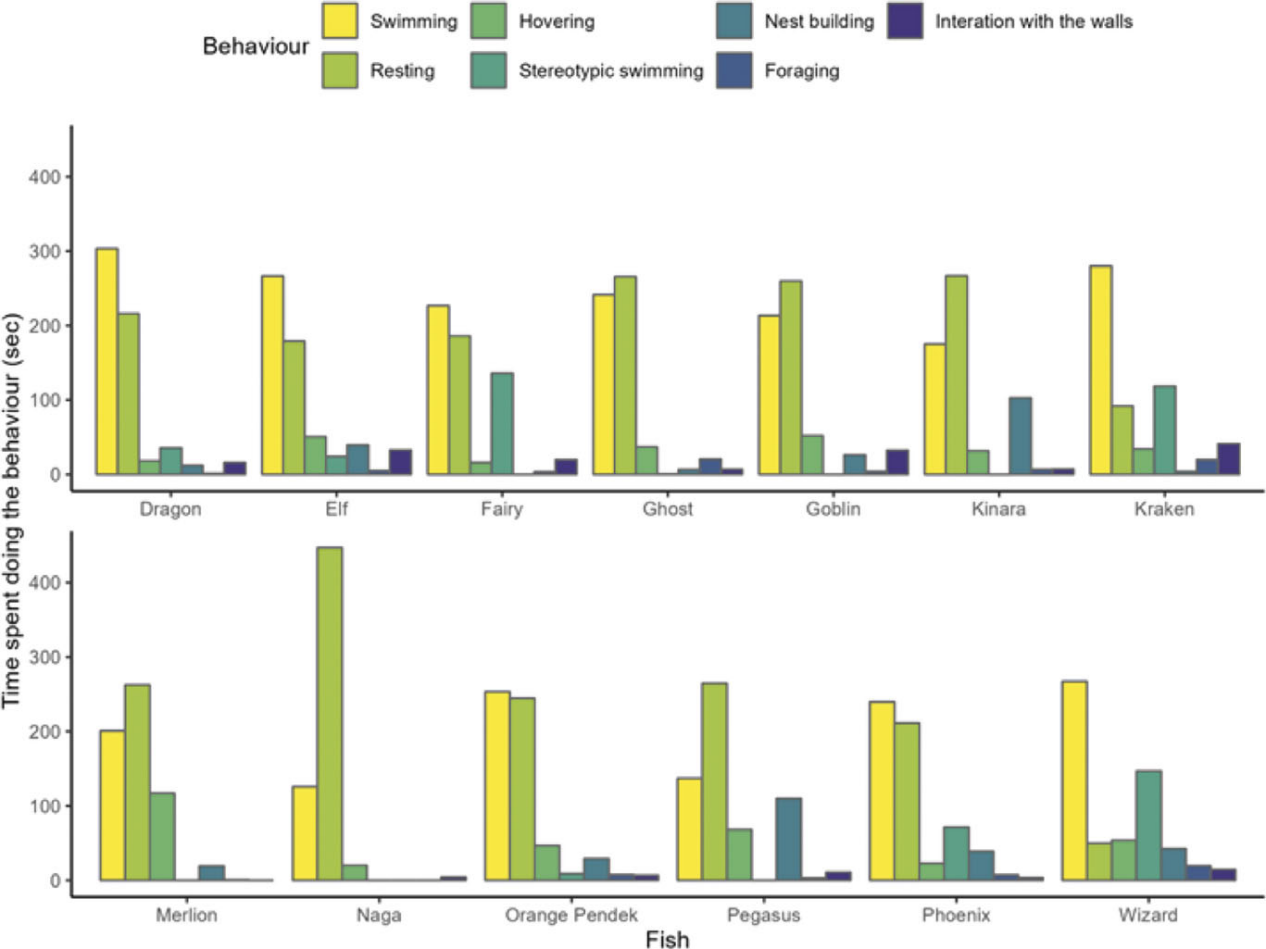
The amount of ime that the Siamese fighting fish (*Betta splendens*; n = 13) engaged in each behaviour across five tank treatments (jar, small, medium, large, large-barren).

**Figure 7:**
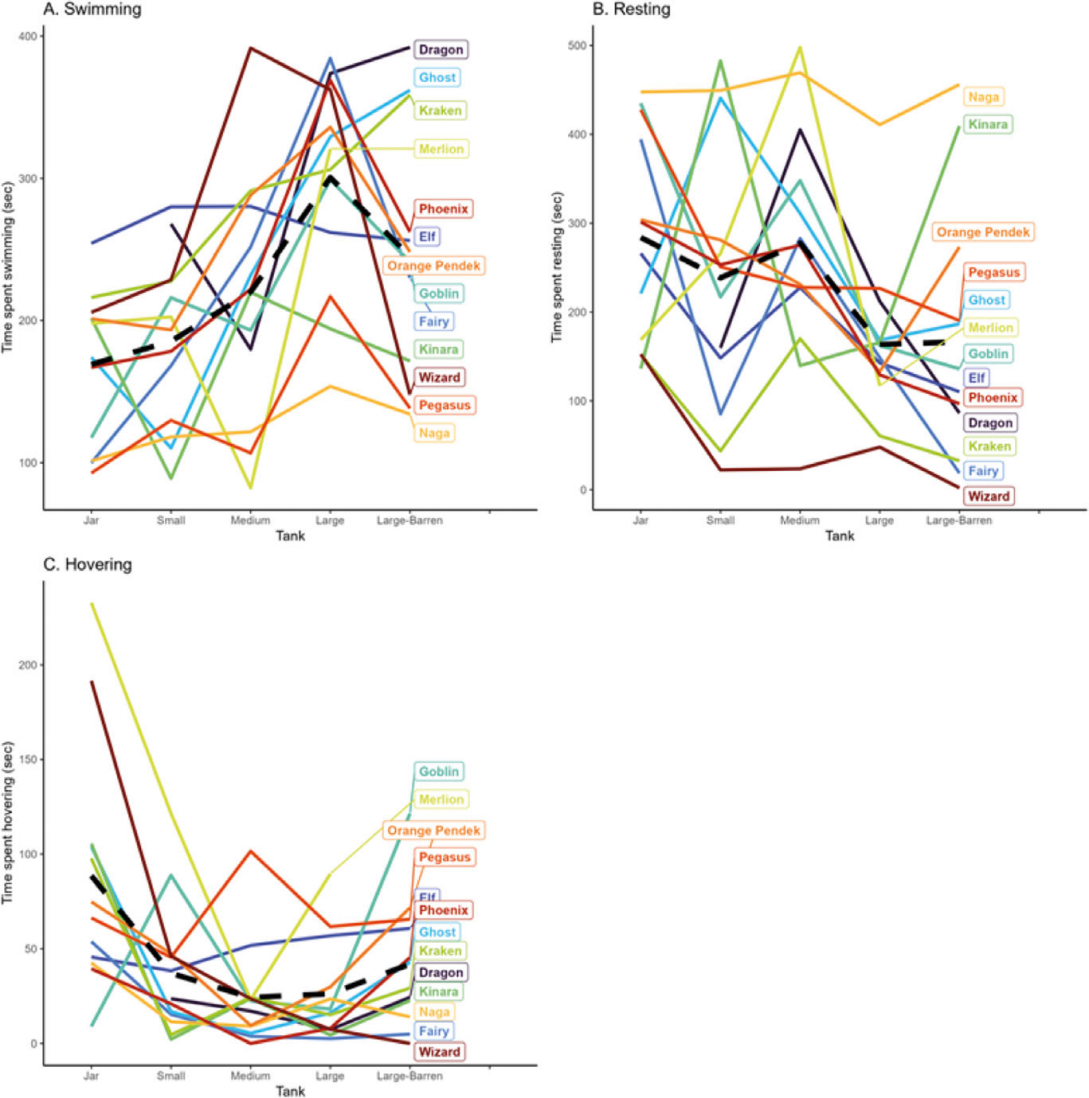
The average amount of time that each of the Siamese fighting fish (*Betta splendens*; n = 13) spent (A) swimming, (B) resting and (C) hovering, across each of the five tank trials (jar, small, medium, large, and large-barren), showing overall trends as well as individual behavioural differences. The dotted black line represents the average across all fish.

The likelihood ratio tests indicate statistically significant random effects for all behaviours, suggesting fish consistently behaved differently from each other (Table 4). Some clear differences include that Naga rested more than other fish, Merlion hovered more than other fish (Figure 6), and Merlion and Ghost rested more on/against plants than other individuals (Figure 5). Wizard, Kraken, Phoenix and Fairy displayed relatively high levels of stereotypic swimming while other individuals such as Pegasus, Naga, Merlion, Kinara, Goblin and Ghost did not perform stereotypic swimming (Figure 6). Further, the type of stereotypic pacing differed; Kraken and Phoenix displayed stereotypic circling, zig-zags and pacing, while Wizard only paced, and Fairy predominantly paced with very little circling. Individual differences, and overall trends, relating to swimming, resting and hovering across tank trials are evident (Figure 7).

## Discussion

Within the pet industry, Siamese fighting fish face particular welfare concerns as they continue to be displayed and kept in small, barren jars (Sermwatanakul 2019). This study aimed to determine if Siamese fighting fish are affected by their housing conditions and, if so, recommend suitable measures to improve their welfare.

We demonstrated that Siamese fighting fish behaviour varied significantly depending on their housing environment. Fish were less active in the jar and small tank, with more resting and less swimming compared to in larger tanks. This finding aligns with a previous study on Siamese fighting fish that also showed reduced swimming in smaller tanks (Oldfield & Murphy 2024). Inactivity potentially signifies negative affective states such as ‘boredom’ in captive animals (Meagher & Mason 2012; Fureix & Meagher 2015). In our study, fish also spent more time displaying behaviours that could be classified as ‘abnormal’ in the smaller tanks compared to the larger tanks; performing higher levels of stereotypic swimming in the small tank, which is a well-acknowledged negative response to captive conditions (Miller *et al.* 2020), as well as higher levels of hovering in the jar. Hovering has been reported as ‘abnormal’ if atypical for the species’ natural behavioural repertoire (Casamitjana 2004). While hovering in Siamese fighting fish can occur during natural nest building (N Clark-Shen, personal observation 2022), this study clearly differentiated the two, with hovering recorded when occurring independently of nest-building activity. Hovering occurred significantly more in the jar than the small tank, and stereotypic swimming occurred significantly more in the small tank than the jar. Based on these observations, we posit that hovering and stereotypic swimming could both represent similar negative reactions to the captive environment (i.e. indicators of poor welfare), but with different expressions possibly driven by space availability (i.e. it is difficult to perform stereotypic pacing where space is particularly limited such as in the jar). Further, hovering was more likely to be performed in the morning, which was also when fish in larger tanks were most active (i.e. swimming), which further supports that hovering may be expressed when fish feel space-constrained and unable to perform more energetic activity. However, hovering also occurred more in the barren tank compared to the large furnished tank, which suggests that hovering may not only take place when space-constrained but as a result of generally less-optimal tank conditions (i.e. an indicator of frustration). In comparison, fish were most active in the large tank, with more swimming and less resting compared to the smaller tanks. Foraging was also exhibited most in the large tank; foraging is considered a natural behaviour indicative of positive welfare (Troxell-Smith *et al.* 2017) and has been reported to occur more in larger, more enriched environments (Mallapur *et al.* 2005). Fish in the large tank also spent the least amount of time engaging in behaviour which could be classified as ‘abnormal’, such as hovering, interacting with the walls and stereotypic swimming. Overall, these findings are similar to other studies which have found increased natural activity among fish in larger tanks (Polverino *et al.* 2016) and increased stereotypic pacing among fish in smaller tanks (Abudusaimaiti *et al.* 2020). Aside from these somewhat expected observations, there were also a number of unexpected findings; for example, nest building occurred most in the small tank (although this was not significant), and stereotypic swimming (a negative behaviour) still occurred in the large tank, despite this tank being considered fairly large in size and enriching in nature; potentially highlighting the difficulty of providing truly optimal tank conditions.

Our study also suggests that tank complexity (i.e. furnishings) is important for welfare; fish performed more ‘abnormal’ behaviours (stereotypic swimming, interaction with the walls and hovering) and less ‘positive’ behaviours (foraging and swimming) in the barren tank compared to in the large tank. As the large and barren tanks were the same size, we can reasonably assume that these differences are in response to the complexity of the tank environment. Other studies of captive mammals (Azevedo *et al.* 2023), reptiles (Bashaw *et al.* 2016), and teleost fishes, sharks and rays (Lawrence *et al.* 2021), have also found a correlation between more enriched environments, increased expression and diversity of natural behaviours and decreased expression of abnormal behaviours. This correlation is ultimately attributed to a more positive experience for the animals (Lawrence *et al.* 2021). Physiological studies support these behavioural findings: fish with no environmental enrichment have been found to have significantly higher cortisol levels and opercular beat rates (implying higher stress) than fish exposed to environmental enrichment (Zhang *et al.* 2020).

To further highlight the importance of tank complexity, we found that when given the choice (e.g. in small, medium and large tanks), Siamese fighting fish spent a considerable amount of resting time (47% of total resting time) on or against furnishings (e.g. plants, hideout). Additionally, all fish used all resting space available to them (ground, surface, plants, barrel) showing that a diversity of resting places could be important. Where and how animals choose to sleep are determined by multiple factors, including protection from predators, visibility of surrounding areas, shelter from weather, and thermoregulation (Anderson *et al.* 2019), and understanding sleeping preferences in the wild can help to elucidate what is needed to improve rest in captivity (Lukas *et al.* 2003; Stewart 2011; Lock 2012). While the sleeping behaviour of wild Siamese fighting fish remains unreported, they are known to live in slow-flowing, shallow waters, often with dense vegetation (Monvises *et al.* 2009), and it is plausible that sleeping amidst vegetation offers protection from predators and anchorage in slow-flowing water. Domesticated Siamese fighting fish may or may not carry this instinctive behaviour (for example, chimpanzees [*Pan troglodytes*] born in captivity build and use nests less than wild-born individuals; Videan 2006), nevertheless, we posit that resting in connection with objects makes them feel less exposed and thus safer. This theory is supported by observations during this experiment involving Merlion, a particularly skittish fish who, upon seeing people (e.g. during feeding), would quickly swim behind a plant, suggesting it offered a sense of safety. Siamese fighting fish may also rest in connection with an object for comfort, which is recognised as a factor in resting choices in great apes and elephants (*Elephas maximus*) (Williams *et al.* 2015; Zamma & Ihobe 2015), or stability: it was observed in this experiment that fish in barren tanks that rested on the ground would ‘flop’ to one side, suggesting that objects (gravel, plants, etc) may help with stability, possibly because of their unnaturally elongated fins. This study on Siamese fighting fish did not record whether when the fish was sleeping on the ‘ground’, they were in contact with the tank walls, but in hindsight this was often observed, and it is worth noting that tank walls could play a similar role as objects within the tank – for example, captive draughtsboard sharks (*Cephaloscyllium isabellum)* show a preference for sleeping in contact with corners of the tank, which they may perceive as safer sleep sites, potentially resembling their resting behaviours in caves and crevices in the wild (Horn 2016; Kelly *et al.* 2021).

### Study limitations

This study only analysed the behaviours of 13 Siamese fighting fish, and more fish would have lent more confidence to the findings. However, ethical considerations influenced the authors to produce results with as low a sample size as possible. Completion of this study with 13 fish saw this aim be achieved, hence more fish were not acquired for the project. Additionally, the order in which the fish experienced each tank differed, and the analysis reveals that this order may have influenced behaviour. Fish spent less time in the barren tank compared to the tanks with furnishings (three days vs seven days), and while this decision was guided by concerns for welfare, a standardised length of time across all tanks would have enabled more robust comparisons. Future studies could consider using a larger sample size and a more standardised rotation of fish through each of the tanks. While the variability of behaviour observed between individuals highlights personality, it also somewhat restricts the ability to generalise findings to the species as a whole and thereby compile species-specific recommendations. Lastly, this study did not examine the optimal ratio of furnishings (i.e. live plants, hideouts) to open space within tanks and so we cannot conclude, for example, that a larger tank with fewer plants would be better for welfare than a slightly smaller tank with more dense plants.

## Animal welfare implications and conclusion

Fishes and other aquatic animals are rarely afforded the same level of compassion as other vertebrates (Brown 2015) with them often excluded from animal welfare legislations around the world (Berlinghieri *et al.* 2021). This study has shown that Siamese fighting fish are affected by their housing environment in captivity and thus greater consideration of their living conditions is warranted.

Based on the behaviours observed in this study, the following are recommended to improve housing conditions for male Siamese fighting fish housed by themselves. For fish on display for sale, a minimum tank size of 5.6 L (‘medium’ in this experiment) is recommended. This recommendation stems from the results which show more swimming and foraging (positive behaviours), and less stereotypic pacing and hovering (negative behaviours) in the medium tank compared to the jar and small tank. While bigger is still better, space and maintenance in fish/pet shops may be a barrier to adoption, hence 5.6 L is likely a realistic compromise. For fish kept as pets, tanks larger than 5.6 L (larger than ‘medium’) are recommended. This recommendation comes from the results which show a decline in hovering and stereotypic swimming (negative behaviours) in the medium tank, and the highest amounts of swimming and foraging (positive behaviours) in the large tank.

When on display for sale, and kept as pets, tanks for Siamese fighting fish should not be barren, but should contain gravel/pebbles, as well as plants and other furnishings (e.g. refuges), to stimulate more natural behaviours and give fish a choice for sleeping and hiding spots. This recommendation comes from the fact that Siamese fighting fish in this study spent a considerable amount of time resting on or against furnishings. Additionally, fish displayed more stereotypic swimming and interaction with the walls (negative behaviours) in the barren tank compared to the large tank which had furnishings. The presence of people is a source of stress for captive animals (Morgan & Tromborg 2007), and so it is imperative that animals in pet shops have the ability to retreat to ‘safe spaces’ if needed.

This study did not look at optimal depth for tanks however, as Siamese fighting fish live in shallow-water environments in the wild, we speculate that tanks that are wider/longer than they are deep, are likely more suitable. Importantly, this study revealed that while general behavioural trends were apparent, differences were observed between individuals, and carers for Siamese fighting fish should pay attention to pets’ personalities to determine what would make for the most optimal tank, for example, relating to preferred sleeping spots (i.e. plants vs hideouts), the boldness of the fish (i.e. shyer fish may need more plants and furnishings to feel safe), and health and age (i.e. sick or older fish may need smaller, shallower tanks as they are less mobile).

## Supporting information

Clark-Shen et al. supplementary material Clark-Shen et al. supplementary material

## Supplementary material

The supplementary material for this article can be found at http://doi.org/10.1017/awf.2024.67.
