# Supplementary material for: Life beyond a jar: Effects of tank size and furnishings on the behaviour and welfare of Siamese fighting fish (Betta splendens)
Source: Anim Welf. 2024 Dec 23;33:e62. doi: 10.1017/awf.2024.67 (PMC11704571; doi:10.1017/awf.2024.67)
Supplement: Clark-Shen et al. supplementary material [file S0962728624000678sup001.pdf]

Supplementary material

Life beyond a jar: Effects of tank size and furnishings on the behaviour and welfare of Siamese fighting fish (*Betta splendens*)

Naomi Clark-Shen[1], *Juliette Tariel-Adam*[2], *Anya Gajanur*[3], *Culum Brown*[2]

[1] Independent researcher, Singapore

[2] Macquarie University, School of Natural Sciences, Australia

[3] National University of Singapore, Department of Biological Sciences, Singapore

Author for correspondence: Culum Brown, email: culum.brown@mq.edu.au

Table S1. Pair-wise contrasts between tanks: Comparison of the size of the difference between tanks for recorded behaviours using contrast estimates, associated *t*- or z-tests, confidence intervals, and *P*-values. Behaviours with a * appended indicate the use of contrast odd ratios instead of estimates, z-test, and asymptomatic upper and lower confidence values to compare differences between tanks. Significant values are shown in bold.

Behaviour	Contrasting tanks		Contrast Estimates	*t*-test/z-ratio	Confidence Intervals		*P*-value
					Lower	*Upper*	
**Swimming**	Jar	Small	-14	-0.9	-56	28	>0.999
	Jar	Medium	-12	-0.69	-59	35	>0.999
	Jar	Large	-93	-5.31	-140	-45	**<0.001**
	Small	Medium	2	0.11	-45	49	>0.999
	Small	Large	-79	-4.53	-126	-32	**<0.001**
	Medium	Large	-81	-5.32	-122	-39	**<0.001**
	Large	Barren	58	3.76	16	101	**<0.001**
**Resting**	Jar	Small	46	2	-17	109	0.186
	Jar	Medium	-4	-0.16	-75	67	>0.999
	Jar	Large	110	4.19	39	181	**<0.001**
	Small	Medium	-51	-1.93	-122	21	0.186

	Small	Large	64	2.43	-8	135	0.080
	Medium	Large	114	5.05	53	175	**<0.001**
	Large	Barren	-5	-0.22	-68	58	>0.999
**Foraging***	Jar	Small	1.88	1.15	0.428	8.250	0.251
	Jar	Medium	0.29	-2.07	0.056	1.459	0.078
	Jar	Large	0.08	-3.97	0.014	0.437	**<0.001**
	Small	Medium	0.15	-2.9	0.027	0.875	**0.015**
	Small	Large	0.04	-4.63	0.006	0.262	**<0.001**
	Medium	Large	0.27	-2.79	0.076	0.952	**0.016**
	Large	Barren	14.64	4.92	3.378	63.486	**<0.001**
**Stereotypic Swimming***	Jar	Small	0.03	-4.56	0.003	0.229	**<0.001**
	Jar	Medium	0.22	-1.59	0.016	2.880	0.284
	Jar	Large	0.18	-1.77	0.014	2.417	0.284
	Small	Medium	7.87	2.39	0.775	79.810	0.100
	Small	Large	6.60	2.20	0.660	66.078	0.138
	Medium	Large	0.84	-0.3	0.171	4.130	0.767
	Large	Barren	0.32	-1.8	0.060	1.743	0.284
**Interaction with surface***	Jar	Small	1.68	1.15	0.503	5.580	0.497
	Jar	Medium	0.69	-0.72	0.174	2.748	0.497
	Jar	Large	4.39	2.80	1.061	18.167	**0.030**
	Small	Medium	0.41	-1.74	0.105	1.625	0.251
	Small	Large	2.62	1.86	0.651	10.547	0.251
	Medium	Large	6.35	3.92	1.783	22.628	**<0.001**
	Large	Barren	0.30	-2.66	0.086	1.012	**0.039**
**Nesting***	Jar	Small	0.29	-2.24	0.065	1.283	1.283
	Jar	Medium	0.25	-2.15	0.046	1.410	1.410
	Jar	Large	0.22	-2.4	0.040	1.205	1.205
	Small	Medium	0.89	-0.22	0.196	3.992	3.992
	Small	Large	0.76	-0.49	0.171	3.398	3.398
	Medium	Large	0.86	-0.27	0.196	3.770	3.770
	Large	Barren	4.17	2.05	0.639	27.143	27.143

Table S2. Results of resting place analysis: Contrasts between the probabilities of resting at the three places. Significant values are shown in bold.

	Estimate	SE	z.ratio	P-value
$log(\frac{P_{resting\ on\ floor}}{P_{resting\ on\ furnishings}})$	0.04	0.038	0.1	0.991
$log(\frac{P_{resting\ on\ floor}}{P_{resting\ on\ the\ water\ surf}}$	4.62	0.365	12.7	**<0.001**

)				
$log(\frac{P_{resting\ on\ furnishings}}{P_{resting\ on\ the\ water\ surf}}$ [illegible])	4.58	0.464	9.9	**<0.001**
